# Trends in Integration Between Physician Organizations and Pharmacies for Self-Administered Drugs

**DOI:** 10.1001/jamanetworkopen.2023.56592

**Published:** 2024-02-19

**Authors:** Pragya Kakani, David M. Cutler, Meredith B. Rosenthal, Nancy L. Keating

**Affiliations:** 1 Department of Population Health Sciences, Weill Cornell Medical College, New York, New York; 2 Department of Economics, Harvard University, Cambridge, Massachusetts; 3 National Bureau of Economic Research, Cambridge, Massachusetts; 4 Department of Health Policy and Management, Harvard T. H. Chan School of Public Health, Boston, Massachusetts; 5 Department of Health Care Policy, Harvard Medical School, Boston, Massachusetts; 6 Division of General Internal Medicine, Brigham and Women’s Hospital, Boston, Massachusetts

## Abstract

**Importance:**

Increasing integration across medical services may have important implications for health care quality and spending. One major but poorly understood dimension of integration is between physician organizations and pharmacies for self-administered drugs or in-house pharmacies.

**Objective:**

To describe trends in the use of in-house pharmacies, associated physician organization characteristics, and associated drug prices.

**Design, Setting, and Participants:**

A cross-sectional study was conducted from calendar years 2011 to 2019. Participants included 20% of beneficiaries enrolled in fee-for-service Medicare Parts A, B, and D. Data analysis was performed from September 15, 2020, to December 20, 2023.

**Exposures:**

Prescriptions filled by in-house pharmacies.

**Main Outcomes and Measures:**

The share of Medicare Part D spending filled by in-house pharmacies by drug class, costliness, and specialty was evaluated. Growth in the number of physician organizations and physicians in organizations with in-house pharmacies was measured in 5 specialties: medical oncology, urology, infectious disease, gastroenterology, and rheumatology. Characteristics of physician organizations with in-house pharmacies and drug prices at in-house vs other pharmacies are described.

**Results:**

Among 8 020 652 patients (median age, 72 [IQR, 66-81] years; 4 570 114 [57.0%] women), there was substantial growth in the share of Medicare Part D spending on high-cost drugs filled at in-house pharmacies from 2011 to 2019, including oral anticancer treatments (from 10% to 34%), antivirals (from 12% to 20%), and immunosuppressants (from 2% to 9%). By 2019, 63% of medical oncologists, 20% of urologists, 29% of infectious disease specialists, 21% of gastroenterologists, and 22% of rheumatologists were in organizations with specialty-relevant in-house pharmacies. Larger organizations had a greater likelihood of having an in-house pharmacy (0.75 percentage point increase [95% CI, 0.56-0.94] per each additional physician), as did organizations owning hospitals enrolled in the 340B Drug Discount Program (10.91 percentage point increased likelihood [95% CI, 6.33-15.48]). Point-of-sale prices for high-cost drugs were 1.76% [95% CI, 1.66%-1.87%] lower at in-house vs other pharmacies.

**Conclusions and Relevance:**

In this cross-sectional study of physician organization–operated pharmacies, in-house pharmacies were increasingly used from 2011 to 2019, especially for high-cost drugs, potentially associated with organizations’ financial incentives. In-house pharmacies offered high-cost drugs at lower prices, in contrast to findings of integration in other contexts, but their growth highlights a need to understand implications for patient care.

## Key Points

**Question:**

What are key trends in the use of physician organization–operated pharmacies?

**Findings:**

This cross-sectional study including 8 million patients found substantial growth in the share of Medicare Part D spending filled at physician organization–operated pharmacies for high-cost, self-administered drugs from 2011 to 2019 including for oral anticancer treatments (from 10% to 34%), antivirals (from 12% to 20%), and immunosuppressants (from 2% to 9%). By 2019, 63% of medical oncologists, 20% of urologists, 29% of infectious disease specialists, 21% of gastroenterologists, and 22% of rheumatologists were employed by organizations operating specialty-relevant pharmacies.

**Meaning:**

Growing physician-pharmacy integration for high-cost drugs highlights the importance of understanding implications for patient care.

## Teaser

This cross-sectional study examines changes in the use of specialty-relevant in-house pharmacies and cost of drugs associated with physician organizations.

## Introduction

Health care organizations across different services have become increasingly integrated in the US. This vertical integration includes integration between hospitals and physicians^1,2^ and skilled nursing facilities^3^ and the integration of physician practices with multispecialty groups,^4^ imaging services,^5^ and ambulatory surgical centers.^6^ Yet, the implications of such integration for cost and quality of care are not fully understood.^7,8,9,10,11,12,13,14,15,16,17,18,19,20,21,22^

One important but understudied margin of vertical integration is between physicians and pharmacy services for self-administered drugs. Physician organizations, including independent practices and health systems, can dispense self-administered medicines directly or via a licensed pharmacy,^23^ and state regulations governing these practices vary. Certain states prohibit dispensing without licensure,^24^ while others prohibit independent physician practices from owning licensed pharmacies (eg, under state antikickback rules).^23,25^ State and federal payers also vary in pharmacy network adequacy rules that may impact the ability of physician organization–based pharmacies to access payer networks.^26^ Nonetheless, industry reports,^27^ expert commentary,^28^ medical guidelines,^29^ surveys,^30^ and numerous case studies^31,32,33,34,35,36,37^ suggest physician organizations are increasingly launching dispensaries and licensed pharmacies. Recent work has also shown substantial growth in colocated pharmacies integrated with oncology practices, also known as medically integrated dispensing.^38^

Still, little is known about trends in physician-pharmacy integration across specialties and drugs and whether drug prices differ at physician organization–operated pharmacies. There is also limited study of the financial factors associated with use of these pharmacies, such as hospital enrollment in the 340B Drug Discount Program, which allows eligible entities to acquire drugs dispensed in-house at 20% to 50% discounts,^39^ generating substantial profit margins. Major growth of the 340B Program may have an important role in physician-pharmacy integration: the share of hospital beds at enrolled hospitals increased from 3% in 1996 to 51% in 2016.^40^ This article addresses these gaps by documenting key trends in the use of physician organization–operated pharmacies across specialties.

## Methods

This study followed the Strengthening the Reporting of Observational Studies in Epidemiology (STROBE) reporting guideline for cohort studies and was approved by the National Bureau of Economic Research Institutional Review Board. The requirement for informed consent was waived because the data were deidentified.

We used Part D and B claims data for a 20% sample of Medicare beneficiaries (calendar years 2011-2019). Each year we limited to beneficiaries enrolled in Medicare fee-for-service Parts A and B continuously or until death and Part D for 1 or more months. The eMethods in Supplement 1 provides details on data cleaning.

We linked Part B claims to the Health Systems and Practice Database (HSPD) to identify claims under common ownership. The HSPD combines data from Medicare, Irving Levin Associates LLC, public reports, and tax filings to link practices (identified by taxpayer identification numbers) and hospitals (identified by Centers for Medicare & Medicaid Service Certification Numbers) owned by common parent entities.^41,42^ We stratified parent entities, referred to as physician organizations, into independent and hospital-linked organizations (ie, owned a hospital) annually. We characterized hospital enrollment in the 340B Program, per prior work.^39^ We used the plurality of evaluation and management claims to annually attribute physicians to organizations and physicians and organizations to a zip code, hospital service area, and state (eMethods in Supplement 1).

We identified physician organization–operated pharmacies, including dispensaries and licensed pharmacies, referred to collectively as in-house pharmacies, each year following an approach similar to that validated in prior work.^38^ First, we identified pharmacies billing Part D for at least $2000 in claims, which accounted for more than 99% of spending. We linked pharmacies to the National Provider and Plan Enumeration System data to identify addresses and organization names. We then identified candidate pharmacies potentially operated by physician organizations. First, as in prior work,^38^ we identified pharmacies sharing an address with a physician, physician practice, or hospital in the National Provider and Plan Enumeration System. This approach may exclude certain specialty pharmacies—often operated by health systems—whose listed addresses were business or distribution centers. To capture such pharmacies, we additionally identified Utilization Review Accreditation Committee–certified specialty pharmacies, which is valued for accessing payer contracts,^43^ and pharmacies for which more than 50% of the spending was prescribed by physicians at one organization. In addition, as in prior work,^38^ we reviewed names, contact information, and websites of all candidates manually to identify pharmacies that appeared truly operated by physician organizations. In-house pharmacies were linked to the physician organization each year whose physicians prescribed the plurality of spending. We then performed several exercises to ensure completeness and specificity in our list including for pharmacies not colocated with a physician, physician practice, or hospital. The eMethods in Supplement 1 provides information on validation checks and examples of pharmacies identified (eTable 1 and eTable 2 in Supplement 1).

We described in-house pharmacy use by drug class for fee-for-service Medicare Part D beneficiaries. In sensitivity analyses, we also examined in-house pharmacy use among all Medicare Part D beneficiaries. Drugs were classified as oral cancer treatments, antivirals, immunosuppressants, or other drugs. Oral cancer treatments were defined per prior work^44^; immunosuppressants and antivirals were defined using Anatomic Therapeutic Classification system classes (immunosuppressants, L04A; antivirals, J05A), excluding oral cancer treatments. Drug molecule cost was estimated using the median annual spending per patient, similar to prior work,^45^ for drugs used by more than 100 patients.

We identified physician organizations in 5 key specialties: medical oncology, urology, infectious disease, gastroenterology, and rheumatology. These specialties were selected because among specialties with a notable share of prescribed spending (>5%) filled at in-house pharmacies, these had the greatest total spending filled at in-house pharmacies in 2019. Physicians’ specialty was identified using the specialty code on the plurality of evaluation and management claims. For each specialty, we identified organizations with any specialists annually; multispecialty organizations could appear in multiple categories. Each year, we defined physician organizations as having a specialty-relevant in-house pharmacy if more than 10% of spending prescribed by its specialists was filled in-house, thus excluding pharmacies in large health systems that are not well integrated with the specialty (eg, hospital retail pharmacies not servicing oncology patients). We showed very similar results using alternative thresholds. For each organization and specialty in each year, we identified all patients with an evaluation and management visit and identified patient characteristics including age, non-Hispanic Black race (based on the Research Triangle Institute algorithm), documented sex, median income in the residence zip code (from the American Community Survey), dual eligibility, urban residence (based on Rural-Urban Commuting Codes), and enrollment in the low-income subsidy program. Race was included in this analysis in order to evaluate differences in patient characteristics between organizations with and without in-house pharmacies and to adjust for patient characteristics in regression analyses.

### Statistical Analysis

Data analysis was performed from September 15, 2020, to December 20, 2023. We compared characteristics of physician organizations with and without in-house pharmacies in 2019. We evaluated unadjusted differences in medians using quantile regression (when not normally distributed) and in means using 2-sample *t* tests (for proportions or when normally distributed). We used multivariate linear regression to evaluate the regression-adjusted association between physician organization characteristics (independent variables) and having an in-house pharmacy (dependent variable). The key organizational characteristics examined were those that may impact the financial viability of pharmacies, including practice size and organization type (independent, hospital-linked, and linked with at least one 340B-enrolled hospital, or hospital-linked and not linked to a 340B-enrolled hospital). We controlled for state fixed-effects and organizations’ mean patient characteristics. We performed sensitivity analyses with a sparser specification (without state fixed-effects or patient characteristic controls) and a richer specification including quadratic terms for all covariates. In regressions, we excluded 3 organizations with missing information on patients’ zip code median income. Pearson testing was used to examine correlation coefficients.

We also compared differences in point-of-sale prices for high-cost drugs (>$10 000 in median annual costs per patient) by pharmacy type in 2019, excluding 136 287 of 6 809 751 claims (2%) that had either outlier prices (>5 times above or below the median drug price each year), fewer than 1 unit dispensed as this may reflect erroneous claims, or missing National Drug Code (NDC) or plan identifiers. We then used a multivariate regression including Part D claims estimating logged point-of-sale prices with an indicator for whether the prescription was filled in-house and fixed-effects for NDC-year-health plan combinations. We also evaluated results under a sparser specification with only NDC-year fixed-effects and a richer specification with hospital service area fixed-effects. We evaluated results by organization type and in an alternative sample of Medicare Advantage patients. Across analyses, 95% CIs excluding 0 were considered statistically significant; Stata, version 17.0 (StataCorp LLC) was used.

## Results

We studied 8 020 652 beneficiaries (median age, 72 [IQR, 66-81] years; 3 450 538 [43.0%] men; 4 570 114 [57.0%] women). Details on sample construction are provided in eTable 3 and patient characteristics are provided in eTable 4 in Supplement 1. From 2011 to 2019, total spending in Medicare Part D increased from $11.5 billion to $19.2 billion (overall), including $330.3 million to $2.3 billion (oral cancer treatments), $448.0 million to $891.2 million (antivirals), $233.7 million to $1.4 billion (immunosuppressants), and $470.7 million to $2.0 billion (other drugs with >$10 000 in median costs per patient).

**Figure 1.**
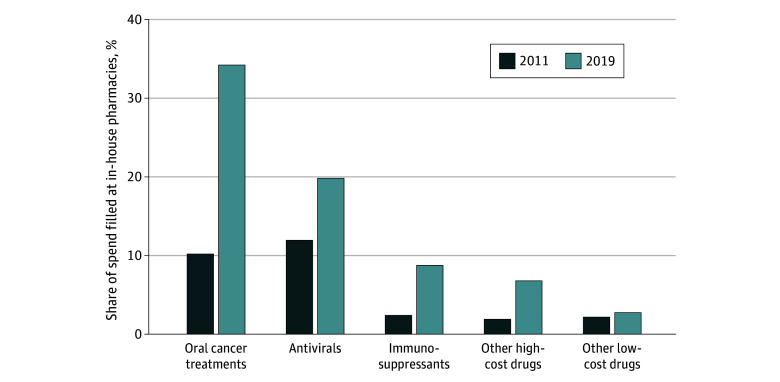
Share of Medicare Part D Spending Filled at In-House Pharmacies By Drug Class (2011-2019) Analysis is limited annually to patients enrolled in Medicare fee-for-service Part A and Part B continuously or until death and Medicare Part D for at least 1 month. Other high-cost drugs (and other low-cost drugs) are drug molecules with more than (less than) $10 000/y median spending per recipient in Medicare Part D over the full study period, excluding cancer treatments, antivirals, and immunosuppressants.

**Figure 2.**
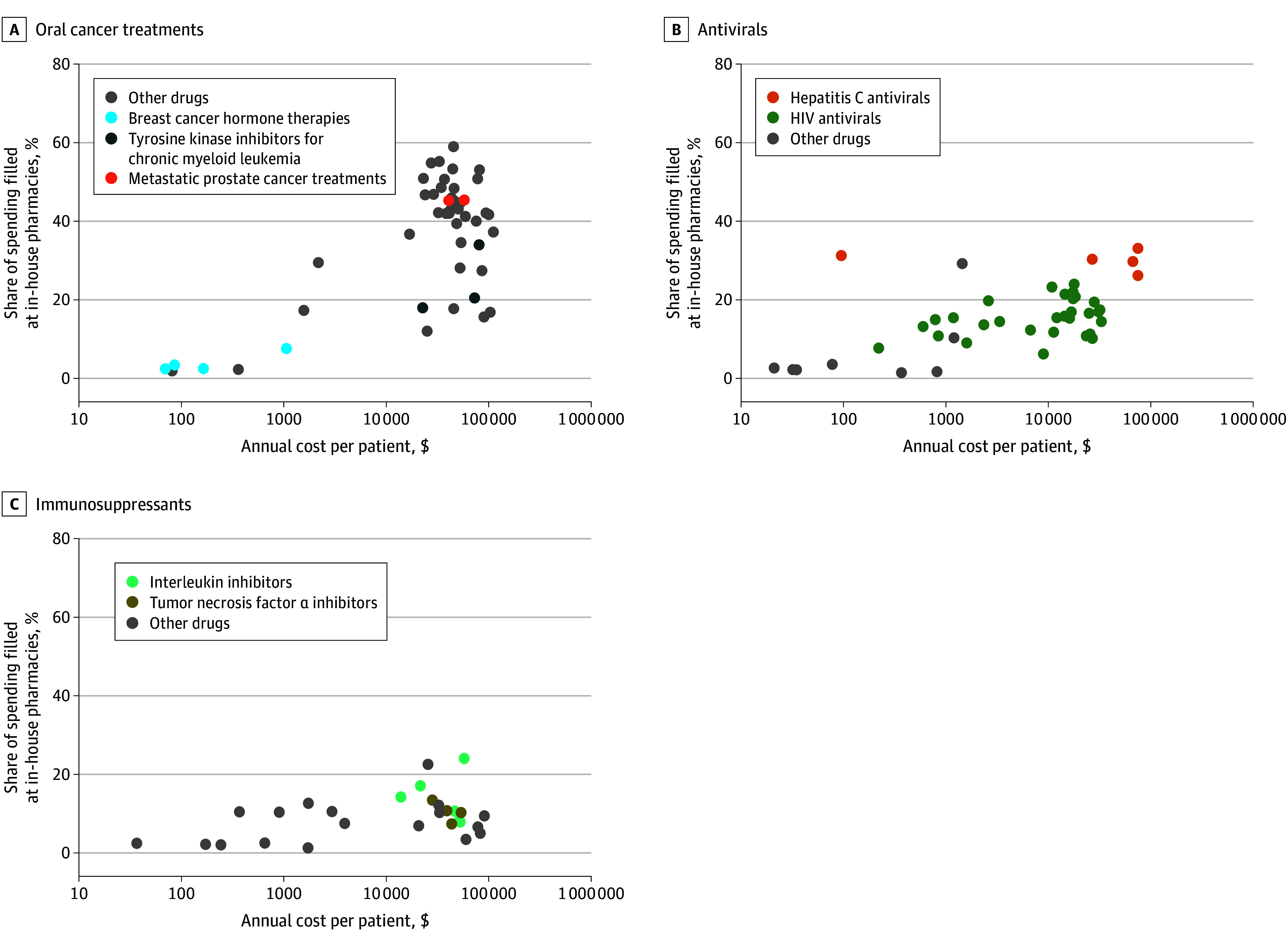
Association Between Drug Cost and Medicare Part D Spending Filled at In-House Pharmacies (2019) Each observation reflects a drug molecule, and the analysis excludes drugs prescribed to fewer than 100 beneficiaries. Annual cost per patient is calculated as the median annual spending on the drug for patients in the analysis sample who were using the drug in 2019. Correlation reported is between share of spending filled at in-house pharmacies and logged annual cost per patient.

From 2011 to 2019, spending at in-house pharmacies increased from $324.1 million (3%) to $1.6 billion (8%) overall, with growth concentrated in high-cost drug classes (Figure 1). Spending filled at in-house pharmacies increased from $33.9 million (10%) to $799.4 million (34%) for oral cancer treatments, $53.7 million (12%) to $176.7 million (20%) for antivirals, $5.6 million (2%) to $126.6 million (9%) for immunosuppressants, and $9.2 million (2%) to $132.9 million (7%) for other high-cost drugs, but was stable for other drugs. Within drug class, higher-cost drugs were more likely to be filled in-house. There was a positive correlation between drug cost and the share of spending filled in-house (*R*^2^ = 0.20; *P* < .001). Figure 2 illustrates this association for oral cancer treatments (*R*^2^ = 0.49; *P* < .001), antivirals (*R*^2^ = 0.32; *P* < .001), and immunosuppressants (*R*^2^ = 0.17; *P* = .03). The average annual point-of-sale spending on high-cost drugs among patients receiving these drugs was $45 924, amounting to an approximate $808 decrease in annual costs per patient associated with in-house pharmacies. This association persisted within physician organization (eTable 5 in Supplement 1), illustrating that these results appear to reflect the choice of organizations with in-house pharmacies to fill primarily high-cost drugs. These findings were similar when evaluated on all Medicare Part D beneficiaries (eFigure and 1 eFigure 2 in Supplement 1).

**Figure 3.**
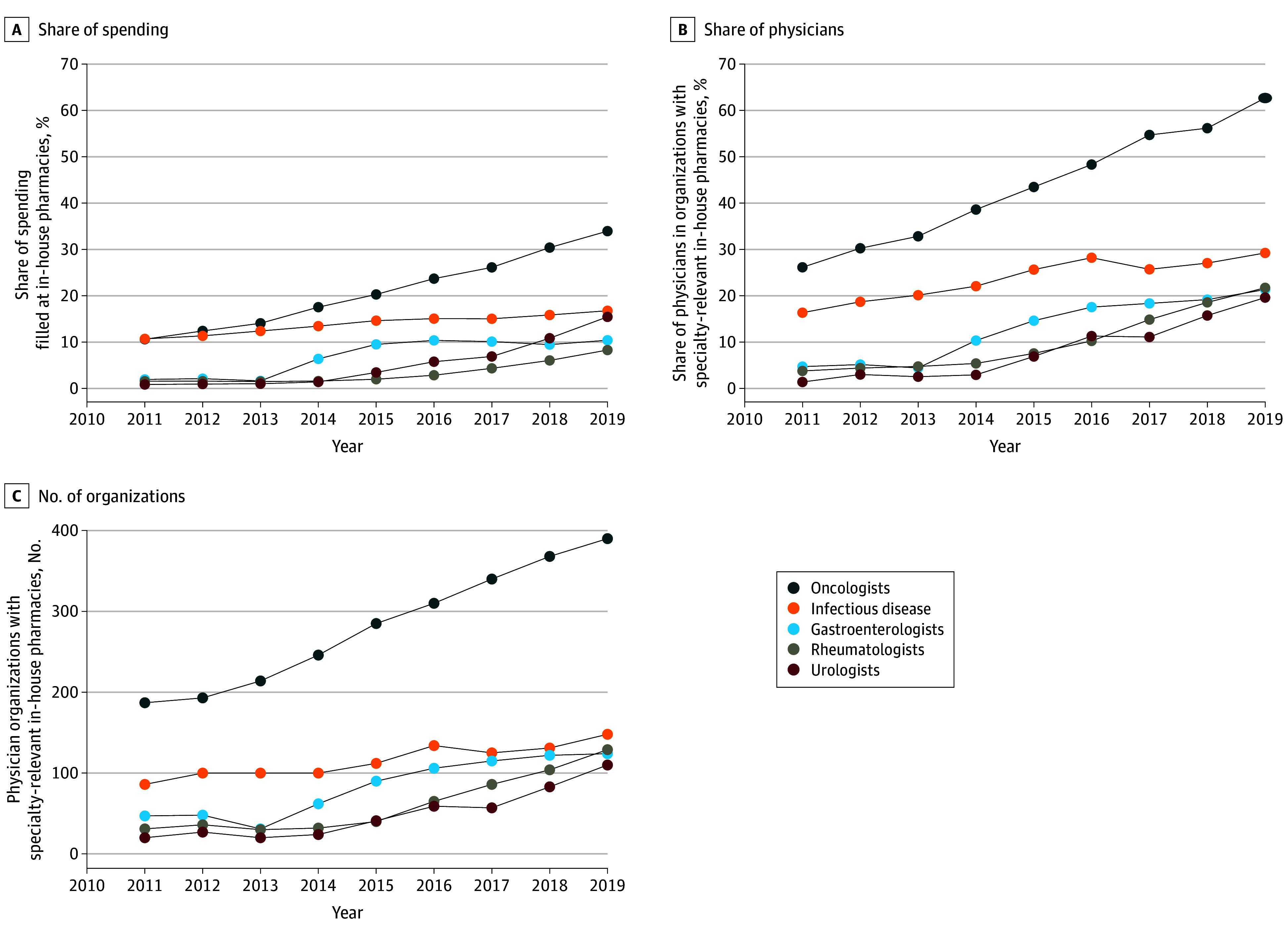
Use of In-House Pharmacies in Oncology, Infectious Disease, Urology, Gastroenterology, and Rheumatology (2011-2019) Medical oncologists were identified using Centers for Medicare & Medicaid Services (CMS) specialty codes 82, 83, 90, and 98. Infectious disease specialists were identified using CMS specialty code 44. Gastroenterologists were identified using CMS specialty code 10. Rheumatologists were identified using CMS specialty code 66. Urologists were identified using CMS specialty code 34. A, Share of spending for medications prescribed by physicians in each specialty that were filled at in-house pharmacies over time. B, Share of physicians in each specialty that was attributed to physician organizations with specialty-relevant in-house pharmacies over time. C, Number of physician organizations in each specialty with in-house pharmacies over time.

The 5 specialties examined experienced growth in the use of in-house pharmacies. The total Medicare Part D spending prescribed increased from $377.6 million to $1.9 billion (oncologists), $92.1 million to $205.3 million (urologists), $190.7 million to $335.6 million (infectious disease specialists), $165.1 million to $451.1 million (gastroenterologists), and $168.1 million to $582.5 million (rheumatologists). Prescribed spending filled in-house increased from $40.1 million (11%) to $661.7 million (34%) for oncologists, $0.8 million (1%) to $31.7 million (15%) for urologists, $20.4 million (11%) to $56.3 million (17%) in infectious disease, $3.2 million (2%) to $47.0 million (10%) for gastroenterologists, and $2.6 million (2%) to $48.3 million (8%) for rheumatologists (Figure 3A). From 2011 to 2019, the total number of physicians increased from 12 306 to 13 799 (oncologists), 9000 to 9265 (urologists), 5213 to 6063 (infectious disease specialists), 12 586 to 13 544 (gastroenterologists), and 4322 to 4706 (rheumatologists). The number of physicians in organizations with in-house pharmacies increased from 3220 (26%) to 8652 (63%) for oncologists, 127 (1%) to 1819 (20%) for urologists, 853 (16%) to 1774 (29%) for infectious disease specialists, 595 (5%) to 2893 (21%) for gastroenterologists, and 164 (4%) to 1023 (22%) for rheumatologists (Figure 3B). The number of physician organizations with in-house pharmacies increased from 187 to 390 (109%) for oncology, 20 to 110 (450%) for urology, 47 to 124 (164%) for gastroenterology, 86 to 148 (72%) for infectious disease, and 31 to 129 (316%) for rheumatology (Figure 3C). These results were similar using alternative definitions of physician-pharmacy integration (eFigure 3 and eFigure 4 in Supplement 1).

Physician organizations with in-house pharmacies differed from other organizations and varied in the share of spending filled in-house in 2019 (Table 1). Across specialties, physician organizations with in-house pharmacies were substantially larger and in 4 of 5 specialties (oncology, infectious disease, rheumatology, and gastroenterology) were more likely to be linked to a 340B hospital. In a pooled analysis across specialties adjusting for patient characteristics, larger organizations had greater likelihood of having a specialty-relevant in-house pharmacy (0.75 percentage point increase for each additional physician [95% CI, 0.56-0.94]), as did organizations with a 340B-enrolled hospital (10.91 percentage point increase [95% CI, 6.33-15.48]). Results were similar in models without state fixed-effects or patient characteristic controls and in models including quadratic forms of all covariates (eTable 6 in Supplement 1). Nonetheless, across all specialties, most physician organizations with in-house pharmacies were not linked to a 340B-enrolled hospital, and most physician organizations linked to 340B-enrolled hospitals did not have in-house pharmacies. Physician organizations with and without in-house pharmacies did not exhibit consistent, large differences in patient characteristics across specialties (eTable 7 in Supplement 1).

**Table 1.** Physician Organization Characteristics Associated With In-House Pharmacies Overall and by Specialty in 2019

Characteristic	Unadjusted differences across organizations			Regression-adjusted association with in-house pharmacies, percentage point change in the likelihood of having an in-house pharmacy (95% CI)^a^
	Organizations with in-house pharmacies, No. (%)	Organizations without in-house pharmacy, No. (%)	Difference, percentage points (95% CI)^b^	
**All specialties combined**				
No. of organizations	901	9836	NA	10 734
No. of physicians in specialty, median (IQR)	8.00 (4.00-20.00)	1.00 (1.00-3.00)	7.00 (6.90-7.10)	0.75 (0.56-0.94)
Organization type				
Independent	369 (40.95)	7751 (78.80)	−37.85 (−40.69 to −35.01)	−3.59 (−6.05 to −1.14)
Hospital-linked and linked to 340B hospital	338 (37.51)	830 (8.44)	29.08 (27.02 to 31.13)	10.91 (6.33 to 15.48)
Hospital-linked but not linked to 340B hospital	194 (21.53)	1255 (12.76)	8.77 (6.45 to 11.10)	[Reference]
Share of drug spending filled in-house, median (IQR), %	39.57 (21.83-62.26)	NA	NA	NA
**Oncology**				
No. of organizations	390	1391	NA	1779
No. of physicians in specialty, median (IQR)	9.00 (4.00-23.00)	1.00 (1.00-3.00)	8.00 (7.27-8.73)	0.66 (0.45-0.87)
Organization type				
Independent	211 (54.10)	943 (67.79)	−13.69 (−19.02 to −8.36)	9.16 (2.94 to 15.38)
Hospital-linked and linked to 340B hospital	112 (28.72)	151 (10.86)	17.86 (13.96 to 21.76)	17.76 (8.90 to 26.62)
Hospital-linked but not linked to 340B hospital	67 (17.18)	297 (21.35)	−4.17 (−8.70 to 0.36)	[Reference]
Share of oncology drug spending filled in-house, median (IQR), %	47.61 (28.18 to 67.41)	NA	NA	NA
**Urology**				
No. of organizations	110	2099	NA	2209
No. of physicians in specialty, median (IQR)	11.00 (5.00-21.00)	1.00 (1.00-3.00)	10.0 (9.81-10.19)	0.97 (0.72-1.23)
Organization type				
Independent	77 (70.00)	1545 (73.61)	−3.61 (−12.08 to 4.87)	1.89 (−0.94 to 4.73)
Hospital-linked and linked to 340B hospital	14 (12.73)	220 (10.48)	2.25 (−3.66 to 8.15)	−5.20 (−8.86 to −1.54)
Hospital-linked but not linked to 340B hospital	19 (17.27)	334 (15.91)	1.36 (−5.67 to 8.39)	[Reference]
Share of urology drug spending filled in-house, median (IQR)	34.30 (18.88-51.69)	NA	NA	NA
**Infectious disease**				
No. of organizations	148	1583	NA	1731
No. of physicians in specialty, median (IQR)	6.0 (2.50-16.00)	1.00 (1.00-2.00)	5.00 (4.91-5.09)	0.89 (0.48-1.29)
Organization type				
Independent	38 (25.68)	1246 (78.71)	−53.04 (−60.00 to −46.09)	−9.23 (−13.39 to −5.07)
Hospital-linked and linked to 340B hospital	70 (47.30)	156 (9.85)	37.44 (32.04 to 42.85)	8.23 (1.04 to 15.42)
Hospital-linked but not linked to 340B hospital	40 (27.03)	181 (11.43)	15.59 (10.01 to 21.17)	[Reference]
Share of infectious disease drug spending filled in-house, median (IQR)	33.41 (18.20-51.74)	NA	NA	NA
**Rheumatology**				
No. of organizations	129	1603	NA	1732
No. of physicians in specialty, median (IQR)	5.00 (2.00-10.00)	1.00 (1.00-2.00)	4.00 (3.88-4.12)	1.07 (0.51-1.63)
Organization type				
Independent	19 (14.73)	1298 (81.97)	−66.24 (−73.25 to −59.24)	−11.25 (−16.64 to −5.86)
Hospital-linked and linked to 340B hospital	71 (55.04)	135 (8.42)	46.62 (41.24 to 52.00)	13.64 (6.24 to 21.05)
Hospital-linked but not linked to 340B hospital	39 (30.23)	170 (10.61)	19.63 (13.85 to 25.40)	[Reference]
Share of rheumatology drug spending filled in-house, median (IQR)	28.85 (18.43 to 59.59)	NA	NA	NA
**Gastroenterology**				
No. of organizations	124	3160	NA	3283
No. of physicians in specialty, median (IQR)	16.00 (6.50-29.00)	1.00 (1.00-3.00)	15.00 (14.84-15.16)	0.57 (0.41-0.73)
Organization type				
Independent	24 (19.35)	2719 (86.04)	−66.69 (−72.95 to −60.43)	−4.98 (−7.77 to −2.19)
Hospital-linked and linked to 340B hospital	71 (57.26)	168 (5.32)	51.94 (47.63 to 56.25)	15.86 (10.10 to 21.62)
Hospital-linked but not linked to 340B hospital	29 (23.39)	273 (8.64)	14.75 (9.58 to 19.91)	[Reference]
Share of gastroenterology drug spending filled in-house, median (IQR)	36.11 (19.6-65.89)	NA	NA	NA

Abbreviation: NA, not applicable.

^a^ Percentage point change was estimated using linear regression models in which observations are physician organizations and organization covariates are used to estimate whether the organization has a specialty-relevant in-house pharmacy; percentage point reflects the coefficient multiplied by 100. Models include the number of physicians in specialty, state fixed effects, and mean patient characteristics including age, mean zip code income, dual eligibility, Black race, and female. Patient characteristics for each organization are based on the mean characteristics of patients with whom the practices’ physicians within specialty have at least 1 evaluation and management visit. Zip code median household income is from the American Community Survey 5-year estimates from 2019 and linked to patients’ zip code of residence. Organizations are attributed to states based on the plurality of their evaluation and management claims each year. Models also include indicators for practice type (independent practices, hospital-linked and linked to a 340B hospital, hospital-linked but not linked to a 340B hospital; the latter is the reference group). Regression models exclude 2 oncology practices and 1 gastroenterology practice with missing data on patients’ median household income. Standard errors are clustered at the state level.

^b^ Confidence intervals for the difference in the median number of physicians in organizations with and without specialty-relevant in-house pharmacies were estimated using a quantile regression in which the outcome is the number of physicians in specialty and the dependent variable is whether the organization has a specialty-relevant in-house pharmacy. Confidence intervals for unadjusted differences in organization type between organizations with and without in-house pharmacies were estimated using a 2-sample *t* test.

Point-of-sale prices paid for high-cost drugs were 1.76% (95% CI, 1.66%-1.87%) lower at in-house pharmacies compared with other pharmacies across drug classes within NDC plan-years (Table 2). Results were similar in models without health plan controls, in models with additional geography controls, when stratified by independent and hospital-linked physician organizations, and when estimated using Medicare Advantage Part D claims (eTable 8 in Supplement 1).

**Table 2.** Association Between In-House Pharmacies and Point-of-Sale Prices for High-Cost Drugs From 2011 to 2019

High-cost drug	Percentage difference in point-of-sale price for drugs filled at in-house pharmacies (95% CI)^a^	No. of observations^b^
Overall^c^	−1.76 (−1.87 to −1.66)	6 673 464
Oral anticancer treatments	−1.12 (−1.22 to −1.03)	1 077 280
Antivirals	−1.91 (−2.07 to −1.76)	2 610 862
Immunosuppressants	−1.36 (−1.57 to −1.15)	1 220 599
Other drugs	−2.45 (−2.86 to −2.04)	1 764 723

^a^ Results of 5 linear regression models in which observations are individual Part D claims for drugs within drug class, the outcome is ln(prices), and the covariates include an indicator for whether the claim was filled at an in-house pharmacy and fixed effects for drug National Drug Code (NDC)–year-plan (all models). The estimate for percent difference in the point-of-sale prices for drugs filled at in-house pharmacies reflects the coefficient on the indicator for whether the claim was filled at an in-house pharmacy. Standard errors are clustered at the NDC-year-plan level. Coefficients are multiplied by 100 to facilitate interpretation as a percent difference.

^b^ Analysis is limited to claims for high-cost drugs, which are defined as drugs with more than $10 000 in median annual costs during the study period, and for patients enrolled continuously in fee-for-service Medicare Part A, Part B, and Part D during the year. Analysis excluded 2% of claims with either outlier prices that were more than 5 times more or less than the median price for claims with the same NDC that year, where fewer than 1 unit was dispensed as this may reflect erroneous claims, missing NDC, or plan identifiers.

^c^ The average annual point-of-sale spending on high-cost drugs among patients receiving these drugs was $45 924, amounting to an approximate $808 decrease in annual costs per patient associated with in-house pharmacies.

## Discussion

There has been substantial growth in integration between physicians and pharmacies for high-cost self-administered medications across multiple drug classes and specialties. This trend has been especially substantial for oral anticancer drugs, where the share of spending filled at in-house pharmacies increased from 10% to 34% between 2011 and 2019 and the share of oncologists at physician organizations with in-house pharmacies increased from 26% to 63%. This estimate is somewhat higher than reported in prior work,^38^ potentially owing to our inclusion of pharmacies whose business offices are not colocated with a practice, as is often true for large health systems. We also observed growth in the use of in-house pharmacies for urologists, infectious disease specialists, gastroenterologists, and rheumatologists, who prescribe high-cost prostate cancer treatments, antivirals, and/or immunosuppressants. Within drug class, in-house pharmacies were substantially more likely to be used for higher- vs lower-cost drugs.

Greater use of in-house pharmacies for high-cost drugs may reflect several factors. First, in-house pharmacies may help patients filling high-cost drugs who experience complexities, such as prior authorization and affordability. Alternatively, profit margins may be greater for high-cost drugs. Pharmacy profit margins may reach 3% to 5% for branded drugs,^46^ a notable amount for drugs with costs that often exceed $100 000 per year.^47^ Because pharmacy market power may have a bigger impact on margins for generic than branded drugs,^48^ in-house pharmacies may achieve lower margins on generic products than chain pharmacies.

The greater adoption of in-house pharmacies among larger physician organizations and those linked to 340B-enrolled hospitals may reflect the economic incentives motivating adoption. Only larger organizations may generate sufficient pharmacy revenue to cover fixed staffing, regulatory, and infrastructure costs and have bargaining power to negotiate sufficiently high reimbursement or low acquisition costs. Consistent with our observation that physician organizations with 340B-enrolled hospitals were more likely to have in-house pharmacies in 4 of 5 specialties, the 340B program may also provide additional incentives for launching such pharmacies. Indeed, 340B-enrolled hospitals can acquire medicines dispensed from within the organizations for up to 20% to 50% discounts^39^ for medications dispensed in-house, generating potentially substantial profit margins. However, the magnitude of this incentive is uncertain due to the increasing use of 340B contract pharmacies. Specifically, 340B-enrolled hospitals can contract with external pharmacies to dispense drugs acquired with 340B discounts, with profits shared.^49^ Thus, hospitals may still earn substantial 340B-related profits without having an in-house pharmacy. This is consistent with our observations that owning a 340B-enrolled hospital is far from perfectly predictive of launching an in-house pharmacy and the association between 340B enrollment and launching in-house pharmacies is not consistent across specialties.

Increasing vertical integration between physician organizations and pharmacies may have important implications for care quality and spending. In other contexts, such as the acquisition of physician practices by hospitals, vertical integration often results in higher prices.^15,16,17,18,19,20,21^ However, we observed that point-of-sale prices for high-cost drugs filled by in-house pharmacies were 1.76% lower than at other pharmacies. Given the average annual point-of-sale spending on high-cost drugs among patients receiving these drugs in our sample was $45 924, this amounts to an approximate $808 decrease in annual costs per patient associated with in-house pharmacies. This may be because launching an in-house pharmacy typically occurs through market entry, rather than through acquisition and thus may be procompetitive. Moreover, individual in-house pharmacies likely have limited market share relative to large chains, potentially reducing market power. In addition, since prescription drug prices and physician services are often negotiated separately, market power in one domain may be less likely to impact prices in another.

Nonetheless, the effects of increasing integration between physician organizations and pharmacies on patient care are poorly understood. On one hand, integration may improve patients’ access to medications and care quality. For example, many patients do not fill newly prescribed high-cost prescriptions^50^ and adherence is suboptimal.^51^ Integration could allow physicians to identify and intervene when patients face do-not-fill prescriptions through reminders, troubleshooting prior authorization, finding financial assistance, or finding alternative treatments. On the other hand, integration may create incentives for overuse. Evidence on infused drugs suggests profitability can impact treatment,^52,53^ and research has found in-house dispensing to be associated with higher drug spending in Switzerland.^54,55,56^ Certain in-house pharmacies may also be less capable of managing care than national firms.^57^

### Limitations

Our study has limitations. First, the analysis may not reflect the commercial market, where regulations governing pharmacy networks differ. Second, similar to other work,^38^ our identification of in-house pharmacies cannot differentiate between pharmacies that were fully owned vs those that were tightly affiliated with the physician organization and ownership was shared with a third party. Third, point-of-sale prices reported in Medicare Part D claims did not include rebates paid or penalties charged to the pharmacy after the point of sale, although from 2013 to 2016 these specific rebates and fees represented less than 1% of gross sales.^58^ We also did not evaluate whether differences in point-of-sale prices translated to lower out-of-pocket costs for patients of in-house pharmacies, which is an important area for future work. In addition, our analysis of characteristics linked with in-house pharmacies is associational. Further research is needed to understand the causal determinants of in-house pharmacy launch, including the impact of government policies such as the 340B Drug Discount Program, regulation of in-house pharmacies, and pharmacy network adequacy rules.

## Conclusions

In this cross-sectional study of physician-pharmacy integration, we found that there has been substantial integration between physician organizations and pharmacies. This trend is likely to continue as increasing financial integration among physician practices increases the share of physicians using in-house pharmacies. Efforts from national firms with pharmacy capabilities (eg, CVS-Aetna, Optum–United Healthcare) to expand primary care service delivery also suggest this trend may expand beyond high-cost drugs. Systematic evaluations of this integration are urgently needed given the growing role of in-house pharmacies in filling self-administered specialty drugs. Understanding the outcomes of integration on care quality and use is key for guiding policy regarding the 340B Drug Discount Program, state regulation governing in-house pharmacies, pharmacy network adequacy rules, and regulatory responses to integration.

## Notes

Supplement 1. **eMethods.** Detailed Methods **eTable 1.** In-House Pharmacy NPIs Accounting for Greatest Medicare Part D Spending at In-House Pharmacies by Specialty in 2019 **eTable 2.** Largest 10 In-House Pharmacies Based on 2019 Part D Spending Filled That Are Not Identified as Co-Located With a Physician, Hospital, or Physician Practice in NPPES Data **eTable 3.** CONSORT Diagram Illustrating Selection of Patient Sample **eTable 4.** Characteristics of Patients and Patient-Years in Sample **eFigure 1.** Share of Medicare Part D Spending on Drugs Filled at In-House Pharmacies by Drug Class in Main Specification and Alternative Sample Including All Medicare Beneficiaries, 2011 to 2019 **eFigure 2.** Association Between Drug Cost and Share of Spending Filled at In-House Pharmacies in Medicare Part D by Drug Class in an Alternative Sample Including All Medicare Beneficiaries (2019) **eTable 5.** Regression-Adjusted Association Between Drug Costliness and Likelihood of Claim Being Filled In-House (2019) **eFigure 3.** Share of Physicians Attributed to Physician Organizations With Specialty-Relevant In-House Pharmacies Using Alternative Definitions of Physician Organization Affiliation With an In-House Pharmacy **eFigure 4.** Number of Physician Organizations With Specialty-Relevant In-House Pharmacies Using Alternative Definitions of Physician Organization Affiliation With an In-House Pharmacy **eTable 6.** Sensitivity Analyses Evaluating the Regression-Adjusted Association Between Select Physician Organization Characteristics and In-House Pharmacies (2019) **eTable 7.** Unadjusted Differences in Mean Patient Characteristics at Organizations With and Without In-House Pharmacies by Specialty (2019) **eTable 8.** Sensitivity Analyses Evaluating the Association Between In-House Pharmacies and Point-of-Sale Prices for High-Cost Drugs (2019)

Supplement 2.
Data Sharing Statement
