# Supplementary material for: Trends in Integration Between Physician Organizations and Pharmacies for Self-Administered Drugs
Source: JAMA Netw Open. 2024 Feb 19;7(2):e2356592. doi: 10.1001/jamanetworkopen.2023.56592 (PMC10877451; doi:10.1001/jamanetworkopen.2023.56592)
Supplement: Supplement 1. — eMethods. Detailed Methods eTable 1. In-House Pharmacy NPIs Accounting for Greatest Medicare Part D Spending at In-House Pharmacies by Specialty in 2019 eTable 2. Largest 10 In-House Pharmacies Based on 2019 Part D Spending Filled That Are Not Identified as Co-Located With a Physician, Hospital, or Physician Practice in NPPES Data eTable 3. CONSORT Diagram Illustrating Selection of Patient Sample eTable 4. Characteristics of Patients and Patient-Years in Sample eFigure 1. Share of Medicare Part D Spending on Drugs Filled at In-House Pharmacies by Drug Class in Main Specification and Alternative Sample Including All Medicare Beneficiaries, 2011 to 2019 eFigure 2. Association Between Drug Cost and Share of Spending Filled at In-House Pharmacies in Medicare Part D by Drug Class in an Alternative Sample Including All Medicare Beneficiaries (2019) eTable 5. Regression-Adjusted Association Between Drug Costliness and Likelihood of Claim Being Filled In-House (2019) eFigure 3. Share of Physicians Attributed to Physician Organizations With Specialty-Relevant In-House Pharmacies Using Alternative Definitions of Physician Organization Affiliation With an In-House Pharmacy eFigure 4. Number of Physician Organizations With Specialty-Relevant In-House Pharmacies Using Alternative Definitions of Physician Organization Affiliation With an In-House Pharmacy eTable 6. Sensitivity Analyses Evaluating the Regression-Adjusted Association Between Select Physician Organization Characteristics and In-House Pharmacies (2019) eTable 7. Unadjusted Differences in Mean Patient Characteristics at Organizations With and Without In-House Pharmacies by Specialty (2019) eTable 8. Sensitivity Analyses Evaluating the Association Between In-House Pharmacies and Point-of-Sale Prices for High-Cost Drugs (2019) [file jamanetwopen-e2356592-s001.pdf]

Supplementary Online Content

Kakani P, Cutler DM, Rosenthal MB, Keating NL. Trends in integration between physician organizations and pharmacies for self-administered drugs. *JAMA Netw Open*. 2024;7(2):e2356592. doi:10.1001/jamanetworkopen.2023.56592

eMethods. Detailed Methods

eTable 1. In-House Pharmacy NPIS Accounting for Greatest Medicare Part D Spending at In-House Pharmacies by Specialty in 2019

eTable 2. Largest 10 In-House Pharmacies Based on 2019 Part D Spending Filled That Are Not Identified as Co-Located With a Physician, Hospital, or Physician Practice in NPPES Data

eTable 3. CONSORT Diagram Illustrating Selection of Patient Sample

eTable 4. Characteristics of Patients and Patient-Years in Sample

eFigure 1. Share of Medicare Part D Spending on Drugs Filled at In-House Pharmacies by Drug Class in Main Specification and Alternative Sample Including All Medicare Beneficiaries, 2011 to 2019

eFigure 2. Association Between Drug Cost and Share of Spending Filled at In-House Pharmacies in Medicare Part D by Drug Class in an Alternative Sample Including All Medicare Beneficiaries (2019)

eTable 5. Regression-Adjusted Association Between Drug Costliness and Likelihood of Claim Being Filled In-House (2019)

eFigure 3. Share of Physicians Attributed to Physician Organizations With Specialty-Relevant In-Houses Pharmacies Using Alternative Definitions of Physician Organization Affiliation With an In-House Pharmacy

eFigure 4. Number of Physician Organizations With Specialty-Relevant In-Houses Pharmacies Using Alternative Definitions of Physician Organization Affiliation With an In-House Pharmacy

eTable 6. Sensitivity Analyses Evaluating the Regression-Adjusted Association Between Select Physician Organization Characteristics and In-House Pharmacies (2019)

eTable 7. Unadjusted Differences in Mean Patient Characteristics at Organizations With and Without In-House Pharmacies by Specialty (2019)

eTable 8. Sensitivity Analyses Evaluating the Association Between In-House Pharmacies and Point-of-Sale Prices for High-Cost Drugs (2019)

eReference

This supplementary material has been provided by the authors to give readers additional information about their work.

© 2024 Kakani P et al. *JAMA Network Open.*

eMethods. Detailed Methods
Section 1: Data Cleaning Procedures

Part B claims (Carrier, Outpatient files) included physician, organization, geography and specialty identifiers, and services rendered. Part D claims included prescriber, pharmacy, drug, and plan identifiers, and the point-of-sale prices paid to pharmacies. We took several steps to standardize the pharmacy and physician identifiers in Part D claims to facilitate our analysis as described below.

<u>Section 1A. Standardizing pharmacy identifiers</u>

Each Medicare Part D claim in our data is associated with a pharmacy identifier. From 2014 onwards, the pharmacy identifier are consistently National Provider Identifiers (NPI). Prior to 2014, the pharmacy identifiers are a combination of NPIs and National Council for Prescription Drug Programs Identifiers (NCPDP IDs). To facilitate standardization, we create an NPI-NCPDP ID crosswalk by combining crosswalks from multiple public data sources.

We begin by using the National Plan and Provider Enumeration System (NPPES) database, which is a database of all healthcare providers, including pharmacies with National Provider Identifiers (NPIs). The NPPES data has optional fields for additional identifiers, such as NCPDP IDs, associated with each NPI. We identify 21,404 NPIs associated with an NCPDP from NPPES. We then complement the NPPES with various online directories of pharmacies from health plan network lists, including historical publicly available health plan network lists. Finally, we find NPIs for 150 NCPDP IDs through other online documentation manually.

The final crosswalk links 95,991 NCPDP IDs to NPIs. This crosswalk allows linkage of over 99% of Part D claims to pharmacy NPIs from 2011 to 2013.

<u>Section 1B. Standardizing physician identifiers</u>

Each Medicare Part D claim in our data is associated with a prescriber identifier. From 2014 onwards, the identifier is consistently an NPI. Prior to 2014, the prescriber identifiers are a mix of National Provider Identifiers (NPIs), Unique Physician Identification Number (UPINs), and Drug Enforcement Administration (DEA) identifiers. We, therefore, standardize prescriber identifiers to NPIs using a variety of sources.

First, we prepare a DEA-NPI crosswalk by combining two sources. We first use the DEA-NPI crosswalk from the National Bureau of Economic Research. We complement this with a DEA- NPI crosswalk prepared by the Centers for Medicare and Medicaid Services from 2010-2012 and currently made available by Black, Viragh, and Moghtaderi (2019).

Second, we prepare a UPIN-NPI crosswalk by combining three sources. The first source is the NBER UPIN to NPI crosswalk available publicly via the National Bureau of Economic Research. This crosswalk is based on the UPIN listed by the physician in his or her NPI application. The second source is the subset of Part B non-institutional physician fees (Carrier file) and Part B institutional claims (Outpatient File) between 2006 and 2008, which have both a UPIN and an NPI listed. In this dataset a UPIN is attributed to an NPI if at least 85% of the claims associated with the UPIN are associated with the same NPI. The final source, provided by Moghtaderi, Viragh, and Black (2019), links physician UPINs to NPIs based on similarity in names in the CMS

UPIN directory and the NPPES NPI directory. In each dataset we limited to UPINs that are affiliated with only 1 NPI. Finally, we dropped less than 5% of UPINs where there was disagreement between the 3 datasets.

These two datasets are able to link the lions' share of Medicare Part D Claims from 2011-2013 to NPIs. In 2011, we are able to link over 99% of Part D claims to a prescriber NPI and the match rate increases to essentially 100% in more recent years.

Section 2. Identifying evaluation and management visits

We link clinicians to organizations and patients to oncologists using a list of all "evaluation and management" visits associated with patients in the main sample as described below; these include all claims for visits with a clinician (e.g., outpatient visits, inpatient visits, emergency room visits, therapy sessions). Using all evaluation and management visits allows the attribution of a variety of providers to provider organizations. This is ultimately important for attributing prescribers in Medicare Part D to provider organizations, specialties, and states.

We use a multi-step process to identify a unique list of all evaluation and management visits. First, we identify the set of CPT billing codes associated with evaluation and management, according to CMS's Berenson-Eggers Type of Service (BETOS) code classification system. Specifically, we create a crosswalk between CPT codes and BETOS codes using physician services claims (Carrier Claims) by attributing each CPT code to the BETOS code associated with the majority of its claims. Approximately 90% of claims for evaluation and management services are accounted for by the 30 CPT codes for office visits[1], hospital care[2], emergency room care[3], ophthalmic services[4], nursing facility care[5], and psychotherapy.[6]

The core challenge in identifying a unique list of evaluation and management visits is that the claims for these visits can appear only once or can be split into two claims (one for the "professional fee" component covering the clinicians' time and one for the "facility fee" component compensating the institution). Most visits are claimed at least once under the professional services component of Medicare Part B claims (the Carrier file), which includes information on the place of service. Visits that occur at facilities such as hospital outpatient departments will also usually have a separate claim for the facility fee component of Medicare Part B Claims (the Outpatient file). However, there are several issues observed in the data. First, on some occasions, a visit that does not occur at a facility can also have a facility fee component billed in the Outpatient file, potentially due to errors in the place of service listed in Carrier claims. The same visit may have slightly different dates in the two files reflecting small differences in the time when the claim was filed. The clinician listed on the facility fee may sometimes reflect a department chair, or another clinician at the institution, rather than the individual actually seeing the patient. Finally, some visits will also only be claimed in the Outpatient file and have no corresponding claim in the Carrier file. This is primarily because some types of facilities receive payment for the professional fee and the facility fee together for outpatient services, including rural health centers, federally qualified health centers, and critical access hospitals. In addition, a small number of institutions eligible to bill professional fees separately do not do so. The reasons for this are not well understood, but some institutions may have poorer management practices that result in no physician service billing for some care.

---

[1] CPT codes 99211, 99212, 99213, 99214, 99215, 99202, 99203, 99204, 99205, G0439
[2] CPT codes 99222, 99223, 99231, 99232, 99233, 99238, 99239, 99291
[3] CPT codes 99283, 99284, 99285
[4] CPT codes 92012, 92014, 92083, 92250
[5] CPT codes 99307, 99308, 99309
[6] CPT codes 90834, 90837

Therefore, we apply an algorithm to capture the unique evaluation and management visits across both the Carrier and Outpatient file, while removing duplicates. For each year, we identify all the claims in the Carrier file that are associated with evaluation and management visits and split these into two samples: claims listed as occurring in a facility that is eligible to claim facility fees in the Outpatient file ("Group A")[7] and those that are not ("Group B"). We then apply the following steps:

- STEP 1: Match Group A claims to Outpatient file evaluation & management claims based on shared beneficiary, day, and physician.
- STEP 2: Match unmatched Group A and Outpatient file claims on beneficiary and day.
- STEP3: Match any unmatched Group A and Outpatient file claims on beneficiary, physician, and day (+/- 7 days).
- STEP 4: For Group B claims and unmatched Outpatient file claims, attempt to match based on beneficiary, physician, and day.
- STEP 5: Retain the Carrier claims for evaluation and management visits + the remaining unmatched Outpatient file claims as the unique set of evaluation and management visits.

In the final set of unique evaluation and management visits each year, 93% of claims are directly from Carrier Claims and 7% of claims come from the Outpatient file only. We then link each evaluation and management claim to its parent organization using the Health System and Practice Dataset (HSPD). For evaluation and management claims deriving from Carrier claims, this is done by linking the tax identifier to the parent organization. For evaluation and management claims deriving from Outpatient claims, this is done by linking the Medicare facility number to the parent organization. Tax identifiers and facility numbers that are un-linkable to a parent organization are considered standalone, independent organizations.

[7] These are identified in the Carrier claims based on place of service codes 19, 22, 23, 53, 49, 72, 62, 65, and 50. This covers hospital outpatient, emergency room visits, community health centers, federally qualified health centers, dialysis facilities, and comprehensive outpatient rehab facilities.

Section 3. Validation checks for identifying in-house pharmacies

We performed two validation exercises to develop confidence in the coverage and accuracy of our list of in-house pharmacies. First, we assessed whether the pharmacies we identified are indeed operated by medical practices. To do this, we selected a random sample of 100 pharmacies and called each to ask whether they are owned and operated by an independent practice or health system. We were able to reach 86 of these pharmacies via phone, with the unreachable pharmacies being ostensibly permanently closed. Of these 86 pharmacies, 83 (97%) were confirmed to be owned and operated by an independent practice or health system. Reasons for residual error included incorrect or incomplete information in NPPES and pharmacy name for an independent pharmacy that is similar to a nearby independent practice or health system.

Second, we assessed whether the pharmacies identified are likely to be a comprehensive set of pharmacies, especially for high-cost drugs. To do this, we identified 2128 pharmacies where at least 75% of their spending is on high-cost drugs with > $10,000 in annual costs over the study period. We manually investigated each of them to identify 1047 that appear to be operated by a physician organization for each year in the data. Of the pharmacy-years represented by these pharmacies in our data, over 99% appeared in our ultimate list of in-house pharmacies. Overall, this suggests that our list is likely a near-comprehensive list of practice-based pharmacies, especially for pharmacies prescribing high-cost drugs. However, it is possible that the list could be missing certain pharmacies owned by practices in certain years, especially those that are owned by practices but not co-located, not primarily serving physicians of a single physician organization, and not certified as a specialty pharmacy. It is also important to note that the precise nature of the financial arrangement between the pharmacy and the practice is unobserved.

See eTable 1 for examples of the largest in-house pharmacies by specialty. See eTable 2 for the 10 largest in-house pharmacies identified that are not co-located with the practice site.

eTable 1. In-house pharmacy NPIs accounting for greatest Medicare Part D spending at in-house pharmacies by specialty in 2019

NPI	Pharmacy Name	Pharmacy or Practice Website
**Oncology**		
1477740298	RX TO GO LLC	https://flcancer.com/oral-oncolytic-pharmacy-rx-to-go/
1700292380	REGIONAL CANCER CARE ASSOCIATES	https://www.regionalcancercare.org/services/pharmacy/
1669519849	TENNESSEE ONCOLOGY	https://tnoncology.com/park-pharmacy/
1851747828	MEMORIAL SLOAN-KETTERING	https://www.mskcc.org/locations/visiting-us/retail-pharmacy
1609970664	MAYO FOUNDATION	https://www.mayoclinic.org/patient-visitor-guide/minnesota/resources/pharmacy-services
**Urology**		
1073918009	INTEGRATED MEDICAL PROFESSIONALS	https://www.aucofny.com/
1528406709	CHESAPEAKE UROLOGY	https://www.chesapeakeurology.com/
1073567905	UROPARTNERS	https://uropartners.com/InOfficeDispensary
1063769701	ACADEMIC UROLOGY OF PA	https://midlanticurology.com/locations/55/academic-urology-care-center
1932289568	UROLOGY OF INDIANA	https://www.urologyin.com/patients/urology-indiana-office-dispensary
**Infectious Disease**		
1083629638	GRADY MEMORIAL	https://www.gradyhealth.org/locations/ponce-de-leon-center/
1629096607	CARES COMM. HEALTH	https://onecommunityhealth.com/locations/midtown-campus/
1073609731	UNIV. OF KENTUCKY	https://ukhealthcare.uky.edu/pharmacy-services
1538226576	JOHNS HOPKINS HOSPITAL	https://www.hopkinsmedicine.org/patient_care/locations/location-results/john-g-bartlett-pharmacy
1962606855	UNIV. OF COLORADO HOSPITAL	https://www.uchealth.org/locations/uchealth-pharmacy-university-of-colorado-hospital-idgp/
**Gastroenterology**		
1073799276	HENRY FORD	https://www.henryford.com/services/pharmacy/delivery
1649507815	CLEVELAND CLINIC	https://my.clevelandclinic.org/departments/pharmacy/services/specialty-pharmacy
1427080415	FAIRVIEW PHARMACY	https://fairviewmnhs.org/Services/Specialty-Pharmacy/
1437253168	UNIV. OF ALABAMA	https://www.uabmedicine.org/specialty-pharmacy-services
1780734103	UNIV. OF CHICAGO	https://www.uchicagomedicine.org/patients-visitors/patient-information/pharmacy
**Rheumatology**		
1346366879	GEISINGER CLINIC	https://www.geisinger.org/pharmacy/locations/geisinger-specialty-pharmacy
1679971485	VANDERBILT	https://www.vanderbilthealth.com/service-line/pharmacy
1144740317	PARTNERS HEALTHCARE	https://www.massgeneralbrigham.org/find-get-care/our-services/specialty-pharmacy
1619031044	UNIV. OF VERMONT	https://www.uvmhealth.org/medcenter/location/pharmacy-1-south-prospect-street
1831461086	UMASS MEMORIAL MEDICAL CENTER	http://www.umsrx.com/contact.php

Source: National Plan & Provider Enumeration System (NPPES) and author's analysis. Note: Pharmacy names are partially abbreviated due to space constraints.

eTable 2. Largest 10 in-house pharmacies based on 2019 Part D spending filled that are not identified as co-located with a physician, hospital, or physician practice in NPPES data

NPI	Pharmacy Name	Pharmacy or Practice Website
1609970664	MAYO CLINIC PHARMACY	https://www.mayoclinic.org/patient-visitor-guide/minnesota/resources/pharmacy-services
1700292380	REGIONAL CANCER CARE ASSOCIATES	https://www.regionalcancercare.org/patients-and-caregivers/patient-services/pharmacy/
1679971485	VANDERBILT UNIVERSITY MEDICAL CENTER	https://www.vumc.org/rx-outpatient/pharmacy-business-office
1427080415	FAIRVIEW SPECIALTY SERVICES PHARMACY	https://mhealthfairview.org/locations/fairview-specialty-pharmacy
1669519849	TENNESSEE ONCOLOGY PLLC	https://tnoncology.com/park-pharmacy/
1225482490	OUTPATIENT PHARMACY SERVICES AT YALE NEW HAVEN HEALTH	https://www.ynhhs.org/patient-care/Outpatient-Pharmacy-Services
1558809756	NYU HOSPITALS CENTER	https://nyulangone.org/locations/nyu-langone-specialty-pharmacy-at-industry-city
1942368352	NEBRASKA MEDICAL CENTER	https://www.nebraskamed.com/pharmacy
1760836381	OSCHNER SPECIALTY PHARMACY	https://www.ochsner.org/locations/ochsner-specialty-pharmacy
1497063895	AURORA SPECIALTY PHARMACY	https://care.aurorahealthcare.org/locations/aurora-mail-order-pharmacy-menomonee-falls

Source: National Plan & Provider Enumeration System (NPPES) and author’s analysis. Note: Pharmacy names are partially abbreviated due to space constraints.

eAppendix 4: Identification of patient sample and patient summary statistics

eTable 3. CONSORT diagram illustrating selection of patient sample

	Patient-year observations	Unique patients across years
20% Random sample of Medicare beneficiaries	104,858,601	16,723,043
Never enrolled in Medicare Advantage during the year[a]	71,058,607	13,215,622
Enrolled continuously or until death in Medicare Parts A & B	60,854,008	11,406,470
Enrolled in Medicare Part D for at least one month	39,653,454	8,020,652
**Final sample**	**39,653,454**	**8,020,652**

Note: [a]Patients who are never enrolled in Medicare Advantage are, by definition, enrolled in Medicare Fee-for-Service.

eTable 4. Characteristics of patients and patient-years in sample

	2011	2019	2011-2019	Latest year
Age, Median	71	72	71	72
IQR	[65, 80]	[67, 79]	[66, 79]	[66, 81]
Female (%)	57.0%	59.5%	57.9%	57.0%
Race				
White (%)	81.0%	82.6%	82.0%	80.4%
Black (%)	11.3%	8.6%	10.1%	10.9%
Other[d] (%)	7.7%	8.7%	7.9%	8.7%
Median Household Income of Zip Code[a], Median Across Patients (000s)	56.7	61.1	59.1	51.9
IQR	[45.7, 75.2]	[48.7, 81.5]	[47.2, 78.8]	[47.2, 78.7]
Ever a full-benefit dual eligible during the year (%)	27.0%	17.9%	21.0%	22.2%
Ever enrolled in the low-Income subsidy during the year[b](%)	44.2%	29.2%	34.8%	36.1%
**Patient – years (N)[c]**	**3,664,224**	**4,688,546**	**39,653,454**	**8,020,652**

Note: [a]Median household income of patients' zip codes are estimated using the American Community Survey 2019 5-year estimates. [b]Low-income subsidy enrollment rates in this sample are higher than enrollment rates across all Medicare beneficiaries primarily because low-income subsidy enrollment was associated with Part D enrollment, and especially so in 2011. [C]Table illustrates characteristics for patient-year observations in 2011, 2019, combined patient-year observations from 2011 to 2019, and for patients' latest year in the data. In 2011, 2019 and for patients' latest year in the data, observations are, by definition, also unique at the patient level. [d]Other race includes Asian/Pacific Islander, Hispanic, American Indian / Alaska Native, and "Other" race categories as calculated by the Research Triangle Institute.

eFigure 1. Share of Medicare Part D spending on drugs filled at in-house pharmacies by drug class in main specification and alternative sample including all Medicare beneficiaries, 2011 to 2019

Panel A. Main Specification

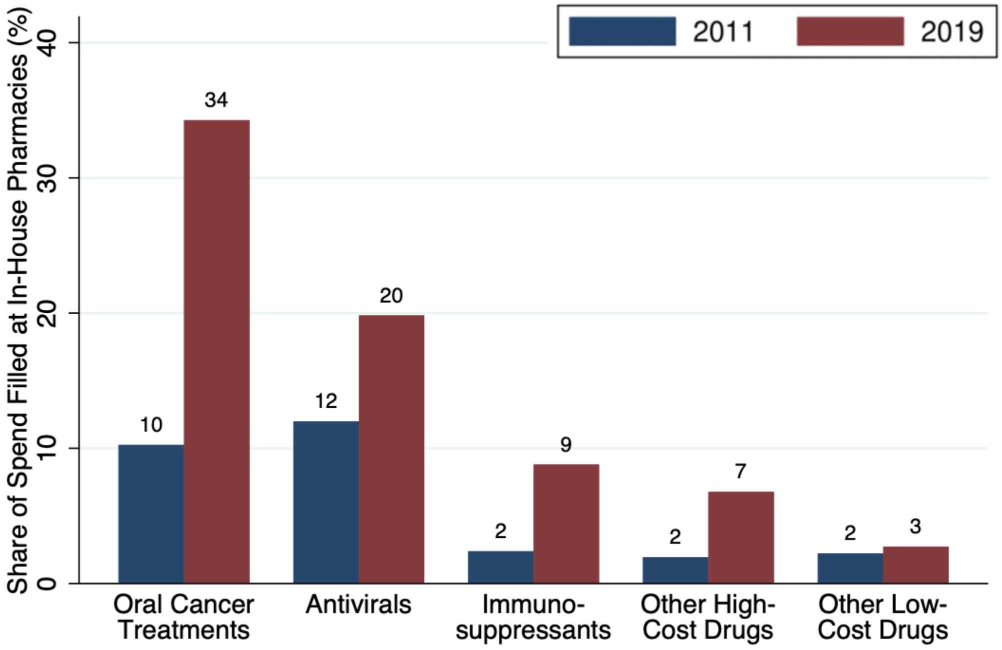

Panel B. All Medicare beneficiaries

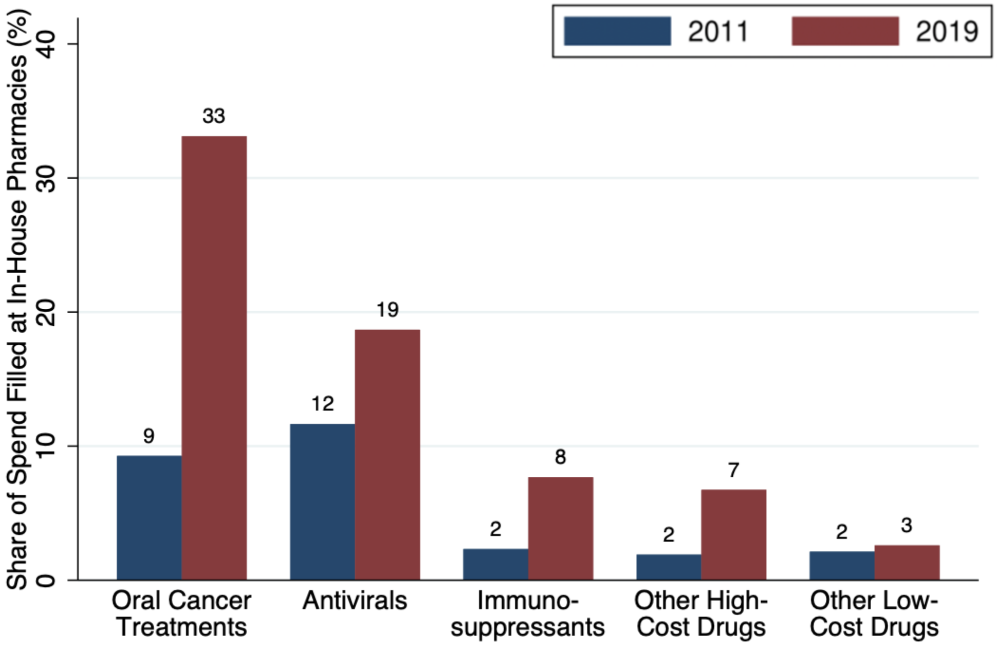

Note: Panel A reflects the results shown in Figure 1 of the main text. Panel B reflects the same analysis but in an alternative specification including all Medicare beneficiaries including those enrolled in Medicare Advantage-Prescription Drug plans.

eFigure 2. Association between drug cost and share of spending filled at in-house pharmacies in Medicare Part D by drug class in an alternative sample including all Medicare beneficiaries (2019)

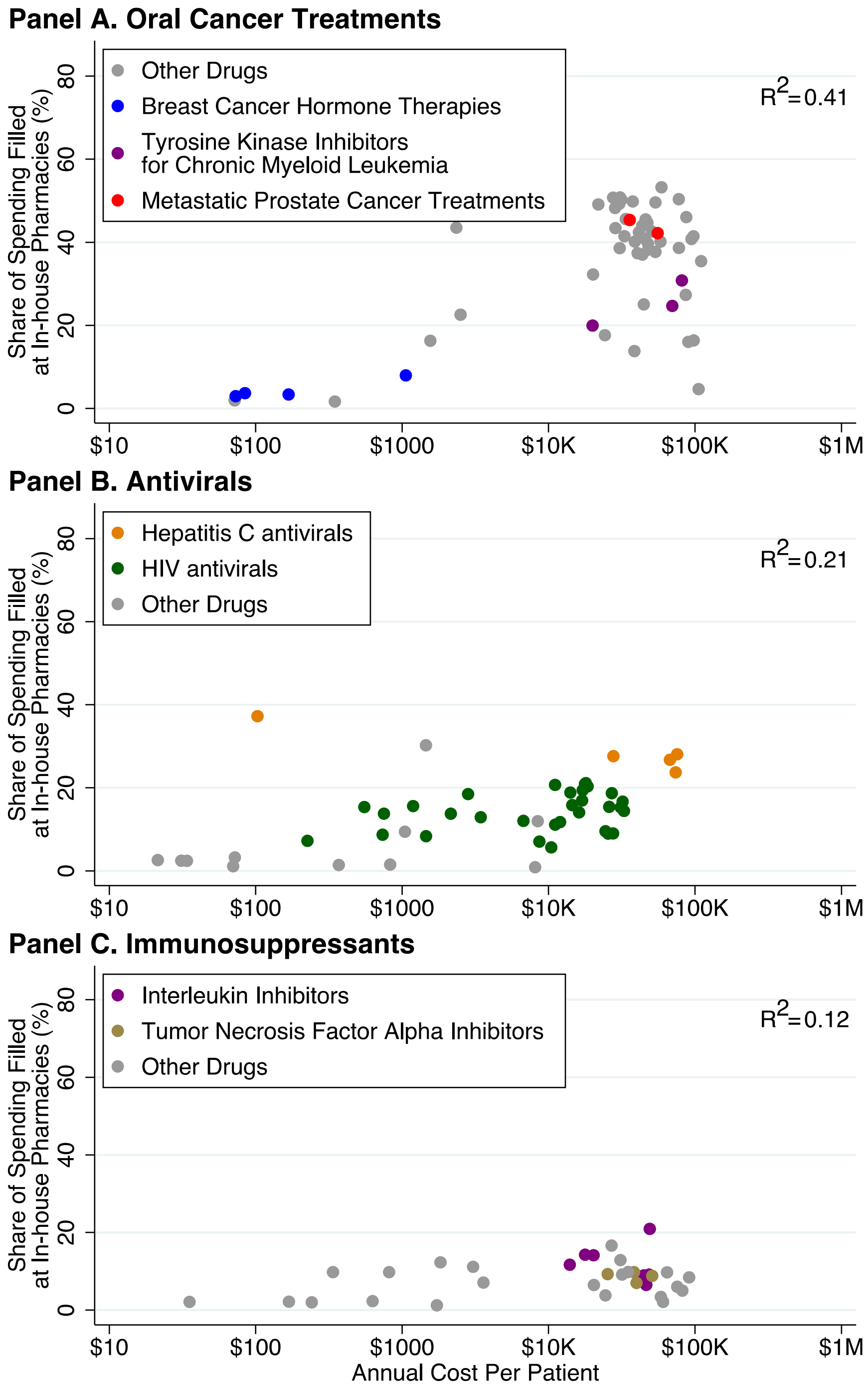

Note: This figure reflects an alternative version of Figure 2 in the main text estimated on an alternative sample including all Medicare beneficiaries, including those enrolled in Medicare Advantage-Prescription Drug Plans.

eTable 5. Regression-adjusted association between drug costliness and likelihood of claim being filled in-house (2019)

Coefficient[a]	Oral Cancer Treatments		Antivirals		Immunosuppressants	
Ln (Annual Drug Cost)	0.05	0.049	0.025	0.021	0.013	0.015
95% CI	(.048, .053)	(.046, .051)	(.023, .026)	(.019, .022)	(.011, .015)	(.013, .016)
N observations[b]	502,655	501,295	870,127	859,143	765,114	761,164
Physician Organization Fixed Effects		X		X		X

Note: [a]This table reflects the results of six linear regression models in which observations are for 2019 Part D claims for drugs in each of the following three categories: oral anti-cancer treatments, antivirals, and immunosuppressants. The model predicts whether the claim was filled at an in-house pharmacy based on the ln(average annual cost of the drug molecule) as defined in the main text. Claims are attributed to the physician organization associated with the prescriber on the claim. Standard errors are clustered at the practice-drug level. [b]Models exclude 4.5% of claims prescribed by a prescriber who is unlinked to a physician organization; these include prescribers that do not have office visits in in Medicare Fee-for-Service Part B claims and thus are not linked to an organization. Models with fixed effects for physician organizations also exclude an additional 1% of claims or claims from physician organizations with fewer than two claims. CI = Confidence Interval.

eFigure 3. Share of physicians attributed to physician organizations with specialty-relevant in-houses pharmacies using alternative definitions of physician organization affiliation with an in-house pharmacy

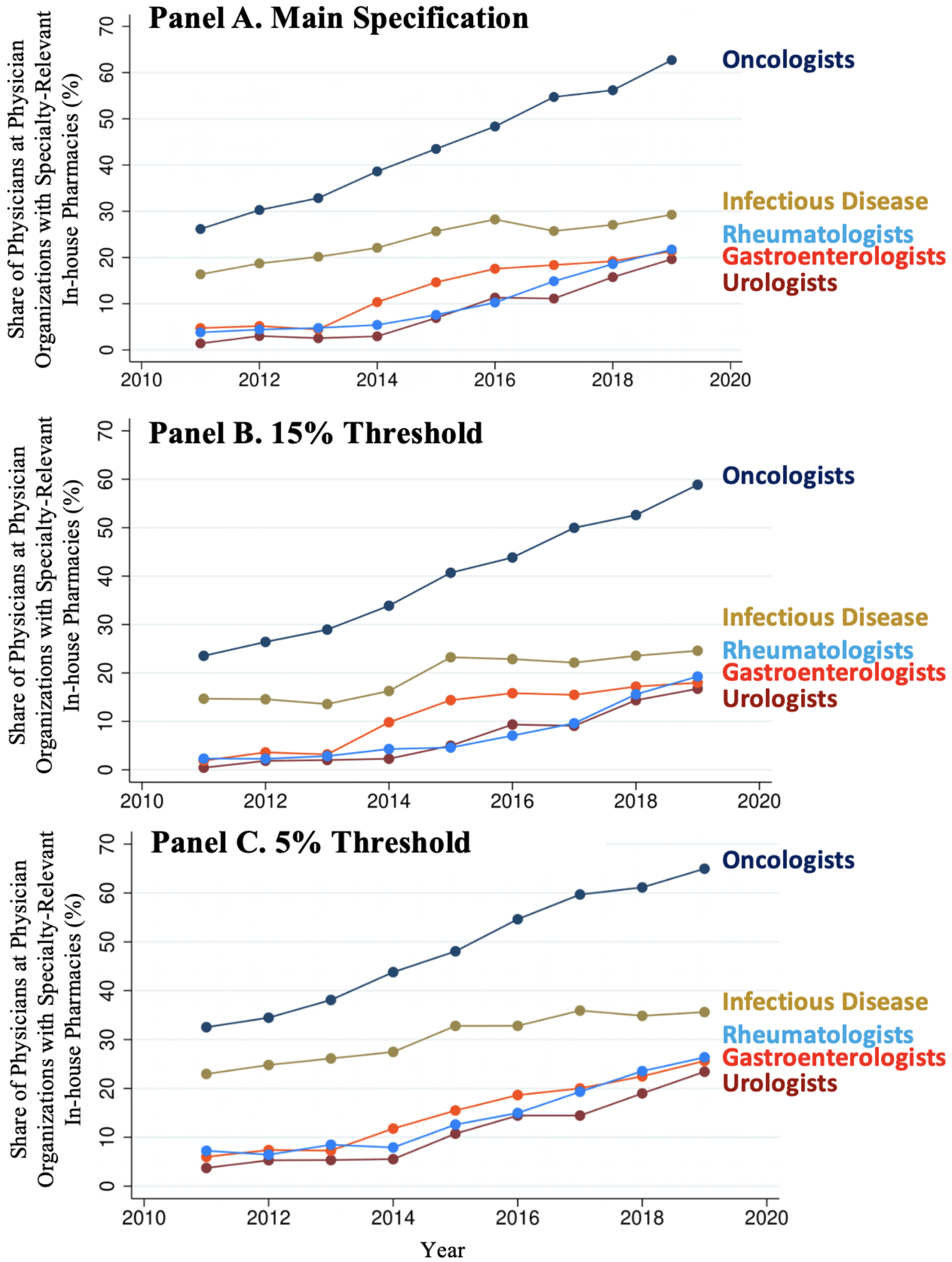

Note: This figure represents alternative versions of Figure 3 Panel B in the main text with alternative definitions of integration. Panel A represents the main specification in which practices are said to have a specialty-relevant in-house pharmacy if at least 10% of spending on prescriptions prescribed by physicians in the specialty are filled by pharmacies attributed to the physician organization. Panel B and C use 15% and 5% thresholds respectively.

eFigure 4. Number of physician organizations with specialty-relevant in-houses pharmacies using alternative definitions of physician organization affiliation with an in-house pharmacy

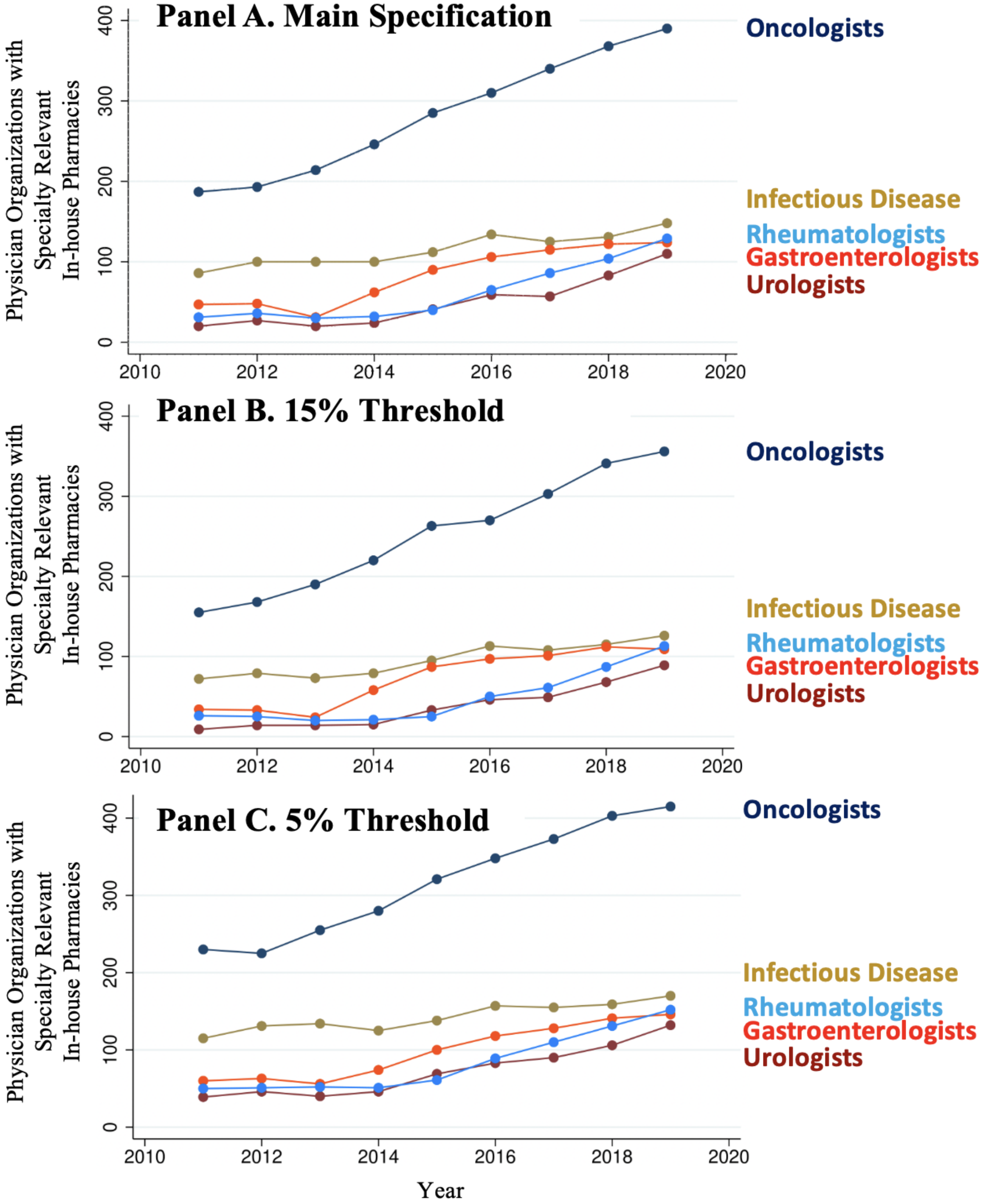

Note: This figure represents alternative versions of Figure 3 Panel C in the main text with alternative definitions of integration. Panel A represents the main specification in which practices are said to have a specialty-relevant in-house pharmacy if at least 10% of spending on prescriptions prescribed by physicians in the specialty are filled by in-house pharmacies. Panel B and C use 15% and 5% thresholds respectively.

eTable 6. Sensitivity analyses evaluating the regression-adjusted association between select physician organization characteristics and in-house pharmacies (2019)

Coefficient[a]	Main Specification[b] (1)	Sparser Specification[c] (2)	Richer Specification[d] (3)
**Panel A. Across All Specialties**			
No of Physicians in Specialty (95% CI)	0.75 (0.56, 0.94)	0.75 (0.62, 0.88)	1.27 (1.11, 1.42)
Physician Organization Type			
Hospital-linked but not linked to 340B hospital	Reference	Reference	Reference
Independent (95% CI)	-3.59 (-6.05, -1.14)	-5.79 (-7.53, -4.05)	-3.66 (-5.50, -1.81)
Hospital-linked and linked to 340B hospital (95% CI)	10.91 (6.33, 15.48)	10.55 (7.43, 13.68)	7.05 (3.95, 10.15)
**Panel B. Oncology**			
No of Physicians in Specialty (95% CI)	0.66 (0.45, 0.87)	0.69 (0.49, 0.89)	1.52 (1.27, 1.77)
Physician Organization Type			
Hospital-linked but not linked to 340B hospital	Reference	Reference	Reference
Independent (95% CI)	9.16 (2.94, 15.38)	4.49 (0.17, 8.80)	11.02 (6.06, 15.97)
Hospital-linked and linked to 340B hospital (95% CI)	17.76 (8.90, 26.62)	16.43 (9.34, 23.53)	8.93 (1.94, 15.91)
**Panel C. Urology**			
No of Physicians in Specialty (95% CI)	0.97 (0.72, 1.23)	1.00 (0.73, 1.26)	1.74 (1.25, 2.23)
Physician Organization Type			
Hospital-linked but not linked to 340B hospital	Reference	Reference	Reference
Independent (95% CI)	1.89 (-0.94, 4.73)	1.18 (-1.39, 3.74)	2.55 (-0.32, 5.42)
Hospital-linked and linked to 340B hospital (95% CI)	-5.20 (-8.86, -1.54)	-5.20 (-9.50, 0.89)	-7.71 (-12.21, -3.21)
**Panel D. Infectious Disease**			
No of Physicians in Specialty (95% CI)	0.89 (0.48, 1.29)	0.90 (0.45, 1.34)	1.95 (1.41, 2.50)
Physician Organization Type			
Hospital-linked but not linked to 340B hospital	Reference	Reference	Reference
Independent (95% CI)	-9.23 (-13.39, -5.07)	-12.07 (-17.21, -6.93)	-7.80 (-12.88, -2.72)
Hospital-linked and linked to 340B hospital (95% CI)	8.23 (1.04, 15.42)	8.17 (0.41, 15.92)	5.2 (-2.56, 12.96)
**Panel E. Rheumatology**			
No of Physicians in Specialty (95% CI)	1.07 (0.51, 1.63)	1.03 (0.54, 1.51)	1.92 (0.86, 2.98)
Physician Organization Type			
Hospital-linked but not linked to 340B hospital	Reference	Reference	Reference
Independent Practice (95% CI)	-11.25 (-16.64, -5.86)	-14.95 (-19.94, -9.96)	-12.39 (-17.56, -7.21)
Hospital-linked and linked to 340B hospital (95% CI)	13.64 (6.24, 12.05)	12.45 (4.03, 20.87)	11.35 (2.73, 19.97)
**Panel F. Gastroenterology**			
No of Physicians in Specialty (95% CI)	0.57 (0.41, 0.73)	0.58 (0.43, 0.73)	0.77 (0.51, 1.04)
Physician Organization Type			
Hospital-linked but not linked to 340B hospital	Reference	Reference	Reference
Independent Practice (95% CI)	-4.98 (-7.77, -2.19)	-6.01 (-9.06, -2.96)	-5.17 (-8.27, -2.08)
Hospital-linked and linked to 340B hospital (95% CI)	15.86 (10.10, 21.62)	15.72 (9.31, 22.14)	14.07 (7.62, 20.52)

Note: [a]Panels A-F reflects the results of individual linear regression models in which observations are physician organizations and organization covariates are used to predict whether the practice has a specialty-relevant in-house pharmacy. [b]The main specification (model 1) is as illustrated in Table 2 Panel B. [c]Model 2 modifies the approach of model 1 by excluding state fixed effects and patient controls. [d]Model 3 modifies the approach of model 1 by testing an alternative functional form which also controls for a quadratic term in all covariates. CI = confidence interval.

© 2024 Kakani P et al. *JAMA Network Open.*

eTable 7. Unadjusted differences in mean patient characteristics at organizations with and without in-house pharmacies by specialty (2019)

Characteristic[a]	Mean patient characteristics in organizations with in-house pharmacies, Median (IQR)	Mean patient characteristics in organizations with in-house pharmacies, Median (IQR)	Difference	95% CI[b]
**Oncology**				
**No. of organizations**	390	1391		
Mean age of patients	73.1 (71.8, 74.2)	73.1 (71.3, 75.0)	0.0	-0.3, 0.3
Percentage of patients of Black race	5.6 (2.1, 12.3)	4.9 (0.0, 15.4)	0.6	-0.9, 2.2
Percentage of patients who were female	62.2 (59.0, 64.9)	62.5 (56.5, 69.2)	-0.4	-1.4, 0.6
Percentage of patients with urban residence	86.8 (70.8, 97.0)	95.1 (68.9, 100)	-8.3	-11.0, -5.7
Percentage of patients who were dual eligible	11.2 (7.7, 17.9)	14.5 (7.3, 25.9)	-3.3	-5.0, -1.7
Mean value of median household income of patients' zip code of residence (000s)[c]	65.5 (56.0, 75.4)	61.7 (51.0, 77.9)	3.8	1.3, 6.4
Percentage of patients ever enrolled in the low-income subsidy benefit	21.4 (15.6, 29.8)	27.1 (15.8, 41.9)	-5.7	-8.1, -3.3
**Urology**				
**No. of organizations**	110	2099		
Mean age of patients	73.6 (72.6, 74.5)	73.9 (72.5, 75.3)	-0.3	-0.8, 0.1
Percentage of patients of Black race	5.7 (1.7, 12.3)	3.1 (0.4 , 8.9)	2.8	1.5, 4.2
Percentage of patients who were female	25.9 (22.2, 29.4)	25.4 (19.5, 30.7)	0.5	-1.4, 2.5
Percentage of patients with urban residence	88.9 (67.6, 97.1)	93.6 (66.7, 99.6)	-4.8	-9.9, 0.3
Percentage of patients who were dual eligible	8.8 (5.7, 14.5)	10.5 (5.3, 20.6)	-1.7	-4.1, 0.7
Mean value of median household income of patients' zip code of residence (000s)	65.9 (56.7, 82.0)	62.6 (51.9, 77.0)	3.4	-1.2, 8.0
Percentage of patients ever enrolled in the low-income subsidy benefit	14.7 (11.4, 22.6)	18.7 (9.8, 31.9)	-3.9	-7.7, -0.2
**Infectious Disease**				
**No. of organizations**	148	1583		
Mean age of patients	67.1 (64.6, 69.5)	71.8 (68.9, 74.9)	-4.8	-5.7, -3.8
Percentage of patients of Black race	12.5 (4.6, 26.5)	8.6 (2.5, 19.4)	4.0	1.2, 6.8
Percentage of patients who were female	46.2 (40.9, 51.3)	51.1 (45.8, 57.0)	-5.0	-6.5, -3.5
Percentage of patients with urban residence	88.1 (68.6, 97.1)	96.2 (84.6, 100)	-8.2	-10.5, -5.9
Percentage of patients who were dual eligible	29.9 (20.8, 39.3)	27.4 (18.1, 41.9)	2.5	-0.9, 6.0
Mean value of median household income of patients' zip code of residence (000s)	59.6 (52.8, 68.5)	63.8 (53.7, 79.1)	-4.2	-8.1, -0.3
Percentage of patients ever enrolled in the low-income subsidy benefit	50.8 (41.3, 64.2)	45.5 (33.1, 60.8)	5.7	1.2, 10.2

Characteristic	Mean patient characteristics in organizations with in-house pharmacies, Median (IQR)	Mean patient characteristics in organizations with in-house pharmacies, Median (IQR)	Difference	95% CI
**Rheumatology**				
**No. of organizations**	129	1603		
Mean age of patients	69.3 (67.2, 70.8)	71.8 (69.7, 73.6)	-2.5	-3.1, -1.9
Percentage of patients of Black race	5.4 (1.4, 14.1)	4.4 (0.9, 11.0)	1.0	-0.6, 2.6
Percentage of patients who were female	73.8 (71.2, 76.6)	75.0 (70.0, 79.5)	-1.2	-2.5, 0.1
Percentage of patients with urban residence	82.5 (61.5, 95.3)	95.0 (78.6, 100)	-12.5	-16.5, -8.6
Percentage of patients who were dual eligible	15.2 (10.2, 24.1)	8.7 (3.3, 18.5)	6.5	4.2, 8.9
Mean value of median household income of patients' zip code of residence (000s)	63.3 (56.6, 71.9)	65.9 (54.9, 83.2)	-2.7	-7.0, 1.7
Percentage of patients ever enrolled in the low-income subsidy benefit	28.0 (19.9, 30.0)	19.0 (9.6, 32.2)	8.9	5.4, 12.5
**Gastroenterology**				
**No. of organizations**	124	3160		
Mean age of patients	69.2 (67.1, 71.2)	72.3 (70.4, 73.9)	-3.2	-3.7, -2.6
Percentage of patients of Black race	8.8 (3.4, 20.9)	4.8 (0.7, 13.1)	4.0	2.1, 5.9
Percentage of patients who were female	57.7 (54.8, 61.3)	59.8 (54.5, 64.6)	-2.1	-3.5, -0.6
Percentage of patients with urban residence	87.2 (70.3, 96.2)	97.6 (84.8, 100)	-10.8	-12.6, -9.0
Percentage of patients who were dual eligible	21.2 (14.8, 32.1)	15.7 (6.9, 28.7)	5.5	2.0, 9.0
Mean value of median household income of patients' zip code of residence (000s)[d]	64.2 (55.8, 73.2)	64.7 (54.3, 82.9)	-0.4	-5.0, 4.1
Percentage of patients ever enrolled in the low-income subsidy benefit	35.9 (27.3, 47.1)	27.3 (14.3, 44.4)	8.8	4.2, 13.4

Note: [a]Table reflects the characteristics of practices with and without in-house pharmacies. For each organization and each measure (age, Black race, female, urban residence, dual eligible, median household income, enrollment in the low-income subsidy benefit) we estimate the mean characteristics of patients with whom the organizations' physicians within specialty have at least 1 evaluation and management visit. Zip code median household income retrieved from American Community Survey 5-year estimates (2019) and linked to patients' zip code of residence. We report the median (IQR) of characteristics across organizations because the distribution of characteristics across organizations is not normally distributed. [b]Confidence intervals for differences in the median characteristic between groups using quantile regression. [c]Two oncology organizations and [d]one gastroenterology organization with missing data on zip code income are excluded from the comparison of median household income of patients' zip code of residence. CI = Confidence Interval; IQR = Interquartile Range.

eTable 8. Sensitivity analyses evaluating the association between in-house pharmacies and point-of-sale prices for high-cost drugs (2019)

	Across classes	Oral Cancer Treatments	Antivirals	Immunosuppressants	Other Drugs
**Panel A. Main Specification[a]**					
Percent difference in point-of-sale price for drugs filled at in-house pharmacies (95% CI)	-1.76 (-1.87, -1.66)	-1.12 (-1.22, -1.03)	-1.91 (-2.07, -1.76)	-1.36 (-1.57, -1.15)	-2.45 (-2.86, -2.04)
N observations	6,673,464	1,077,280	2,610,862	1,220,599	1,764,723
**Panel B. Model Only Controlling for NDC-Year Fixed Effects[b]**					
Percent difference in point-of-sale price for drugs filled at in-house pharmacies (95% CI)	-1.70 (-1.84, 1.56)	-1.04 (-1.27, -0.82)	-1.98 (-2.16, -1.81)	-1.39 (-1.70, -1.09)	-2.23 (-2.80, -1.66)
N observations	6,673,464	1,077,280	2,610,862	1,220,599	1,764,723
**Panel C. Model Additionally Controlling for Hospital Service Area Fixed Effects[c]**					
Percent difference in point-of-sale price for drugs filled at in-house pharmacies (95% CI)	-1.75 (-1.86, -1.65)	-1.15 (-1.26, -1.03)	-1.91 (-2.06, -1.76)	-1.06 (-1.25, -0.87)	-2.57 (-2.94, -2.20)
N observations	6,279,482	1,015,593	2,443,851	1,169,906	1,649,881
**Panel D. Model evaluated for an alternative patient sample enrolled in integrated MA-PD plans[d]**					
Percent difference in point-of-sale price for drugs filled at in-house pharmacies (95% CI)	-1.72 (-1.87, -1.56)	-0.69 (-0.93, -0.44)	-1.99 (-2.22, -1.77)	-1.81 (-2.14, -1.47)	-1.74 (-2.02, -1.46)
N observations	3,516,853	603,910	1,290,931	776,973	845,039
**Panel E. Evaluation of effects for independent and health system-owned pharmacies in primary sample[e]**					
Percent difference in point-of-sale price for drugs filled at in-house pharmacies attributed to independent practices (95% CI)	-1.30 (-1.41, -1.18)	-0.72 (-0.82, -0.62)	-1.45 (-1.60, -1.30)	-2.03 (-2.56, -1.50)	-1.38 (-1.95, -0.81)
Percent difference in point-of-sale price for drugs filled at in-house pharmacies attributed to hospital-linked practices (95% CI)	-2.18 (-2.35, -2.02)	-1.64 (-1.79, -1.49)	-2.32 (-2.56, -2.07)	-1.15 (-1.37, -0.93)	-3.51 (-4.05, -2.97)
N observations	6,673,464	1,077,280	2,610,862	1,220,599	1,764,723

Note: [a]Panel A replicates the results of Table 2. [b]Panel B reflects results using a sparser set of controls; specifically Panel B only controls for NDC-Year combination. [c]Panel C reflects results using a richer set of controls and includes controls for NDC-Year-Plan combination as well as fixed effects for Health Service Area (HSA). Models with Hospital Service Area fixed effects exclude claims written by prescribers who were not linkable to a Hospital Service Area (5.8% of claims); this occurs for prescribers who do not have Medicare fee-for-service evaluation and management visits with patients in our sample during the year and thus cannot be linked to a zip code or because they are linked to a zip code that is not attributed by the Dartmouth Atlas to a Hospital Service Area. [d]Panel D evaluates the model on an alternative 20% sample of Part D claims made for patients in integrated Medicare Advantage – Prescription Drug (MA-PD) plans. [e]Panel E is a variant of the main analysis wherein separate effects are estimated for independent practices that are not co-owned with a hospital and hospital-linked practices, which are co-owned with a hospital. Standard errors in Panel A, C, D, and E are clustered at the NDC-Year-Plan level. Standard errors in Panel B are clustered at the NDC-Year level. CI = confidence interval.

eReference

Moghtaderi, A., Viragh T., Black, B. Overview of Master Individual Provider Crosswalk. American Society of Health Economics Annual Meeting. 2019. Available here: https://ashecon.confex.com/ashecon/2019/webprogram/Paper7938.html
