# Supplementary material for: Trends in Integration Between Physician Organizations and Pharmacies for Self-Administered Drugs
Source: JAMA Netw Open. 2024 Feb 19;7(2):e2356592. doi: 10.1001/jamanetworkopen.2023.56592 (PMC10877451; doi:10.1001/jamanetworkopen.2023.56592)
Supplement: Supplement 2. — Data Sharing Statement [file jamanetwopen-e2356592-s002.pdf]

Data Sharing Statement

Kakani. Trends in Integration Between Physician Organizations and Pharmacies for Self-Administered Drugs. *JAMA Netw Open*. Published February 19, 2024. doi:10.1001/jamanetworkopen.2023.56592

Data
Data available: No

Additional Information
Explanation for why data not available: This study relies primarily restricted Medicare Research Identifiable Files (RIFs) from the Centers for Medicare and Medicaid Services (CMS). Our data use agreements prohibit sharing of these data directly, but these data can be acquired by other researchers and detailed information on procedures for replicating our results are included herein and in the Supplementary Materials. Analytic code will also be made available upon request to the corresponding author.
